# Modified Buttermilk in Infant Feeding

**Published:** 1907-06

**Authors:** C. F. Judson, R. O. Clock

**Affiliations:** Philadelphia; Philadelphia


### MODIFIED BUTTERMILK IN INFANT FEEDING.*

By C. F. Judson, M.D., and R. O. Clock, M.D., Philadelphia.

Buttermilk has recently taken its place as a legitimate food for infants. For the past twenty years foreign pædiatrists have given this method of feeding their serious attention. J. L. Morse was one of the first American writers to call attention to the value of buttermilk, in a paper published in the Archives of Pædiatrics. Notwithstanding, comparatively few pædiatrists have recognized the advantages pertaining to buttermilk diet in certain forms of disease. Moreover, the prejudice against this form of feeding in the minds of many mothers is deep seated and often cannot be overcome.

The chief practical difficulty is to procure a fresh buttermilk from daily churnings. Some of our daries now furnish a reliable product. In the second place, it is not an easy matter to heat buttermilk to the boiling point withou coagulaion of the casein in large lumps, too large to pass through the nipple. A small quantity of wheat or barley flour is added for the purpose of preventing this clotting; it must be gradually sifted, with constant stirring.

The buttermilk fed to the twelve infants which I report by no means met all the requirements of an infant food. The dairyman churned but twice a week, so that the buttermilk when it reached the hospital was often two days old. The degree of acidity was not determined, it varied considerably from day to day. The buttermilk was kept on ice from the time of its preparation until it reached the hospital.

The average composition of whole buttermilk (according to Salge) is proteid 2.5 to 2.7 per cent., sugar 3 to 3.5 per cent., and fat 0.5 to 1 per cent. To this some sugar is added to bring the proportion of sugar up to 7, or 8, or even 10 per cent.; besides a small quantity of wheat flour, and if desired small amounts of cream, can be incorporated in the mixture. It is then heated to the boiling point for a few moments, cooled, and kept on ice until used. This is the usual mode of preparation recommended by foreign pædiatrists.

In preparing the buttermilk for these cases two deviations were made from the usual method. The casein was diluted considerably to make the proportion present 1.5 to 2 per cen., and the sugar added only in sufficient amounts to bring the proportion present in the mixture up to 5 per cent. Moreover, the mixture was not brought to the boiling point, but heated only to from 140 to 155 degrees F. for ten minutes, so that the lactic acid bacteria were not destroyed. Robinson's barley flour was added instead of wheat flour, one-half ounce to each pint and a half, and cane sugar solution (6 and 9 per cent. strength) used to dilute the casein. The method of preparation of the weaker mixture was as follows: Ingredients used were one pint of buttermilk, eight ounces of a six or nine per cent.

solution of cane sugar, one-half ounce of Robinson's patent barley.

1. Make a paste of the flour and a small quantity of the sugar solution.

2. Add buttermilk and the remainder of the sugar solution to this paste and mix thoroughly.

3. Heat the mixture to 155 degrees F. for ten to fifteen minutes, stirring constantly.

4. Remove from stove, cool, and place on ice.

In feeding the infants on buttermilk the principle followed was to begin with a moderately strong mixture, which we called "two-thirds" mixture, containing 1.0 to 1.5 per cent. fat, 4 to 5 per cent. sugar, and 1.5 to 1.75 per cent. proteid. After tolerance for this mixture had been established, as evidenced by normal temperature and good condition of the stools, the fat percentage was increased. If the infant's digestion still remained good, the formula was increased to "three-fourths" mixture with a simultaneous increase in the fat percentage. The three-fourths mixture contained 1.5 to 2.5 per cent. fat, 5 per cent. sugar, and 1.75 to 2.00 per cent. proteid.

The twelve infants treated were severe cases of malnutrition, marasmus, enteritis, or enterocolitis. The prostration and other toxic symptoms quickly disappeared in most cases after buttermilk diet was instituted. Eight infants gained in weight (in one the gain was only temporary), while four infants could not tolerate the diet. The gain in weight was in most cases irregular, but steadily upward, and averaged one-half ounce daily. Some gained more rapidly for shorter periods.

The longest period of continuous feeding on buttermilk was six weeks. During this time no evidences of rachitis developed; on the contrary, the muscles became firm and the skin lost its dryness. The color improved, and the infants were quiet and happy and slept well.

The appearance of the fæces of infants on buttermilk diet was at first brown and pasty, and very offensive; later the evacuations became yellowish brown, and finally yellow. The stools contained as a rule mucus, showing the catarrhal inflammation of the intestines.

After buttermilk diet was instituted, the improvement in the intestinal condition was shown by the disappearance of mucus from the stools and the absence of offensive odor.

From our limited experience with buttermilk feeding, its advantages appear to us to consist principally in that we can give a sufficient amount of proteid to these cases of wasting (1.5 to 2.5 per cent.), and that the casein lactate in the mixture is in a finely subdivided form which admits of easy digestion. The low fat content of the mixture (1 to 2 per cent. does not seem a disadvantage, since many of these infants show an intolerance for fat. Moreover, buttermilk feeding for our infants was used as a temporary feeding, to bridge the way, after satisfactory gains had been made, to corresponding milk formulæ. The proportions of sugar in our formulæ, (4 to 5 per cent.) are distinctly low, and more decided gains in weight might have been obtained with higher sugar content (7 to 8 per cent.). In any case the results obtained were sufficiently favorable to encourage us to pursue this mode of feeding further.

The degree of heat applied to the buttermilk did not exceed 155 degrees F. (140 to 155 degrees F.), so that the lactic acid bacilli were not destroyed. The bacterial flora in the fæces were probably modified, but unfortunately we could not carry out this line of investigation.

Comparing the results of buttermilk feeding in the class of cases mentioned with the results obtained from milk formulæ, and whey and cream mixtures the results are certainly equally good. This must be ascribed chiefly to the large amount of soluble proteid furnished in finely sub-divided form. The proportion of soluble albumin to casein is also somewhat greater than in plain milk.

From our limited observation we consider that buttermilk feeding should not as a rule be continued as the exclusive food for an infant, even with the addition of cream, for a longer period than eight to twelve weeks; but the attempt should be made as soon as the child's condition permits to change over to a milk mixture of corresponding strength.—N. Y. Med. Jour., April 20th, '07.

